# Lipotoxicity: a driver of heart failure with preserved ejection fraction?

**DOI:** 10.1042/CS20210127

**Published:** 2021-10-13

**Authors:** Jennifer Leggat, Guillaume Bidault, Antonio Vidal-Puig

**Affiliations:** Metabolic Research Laboratories, Wellcome Trust-MRC Institute of Metabolic Science, Addenbrooke’s Hospital, Cambridge, CB2 0QQ, U.K.

**Keywords:** diastolic dysfunction, heart failure with preserved ejection fraction, lipid metabolism, lipotoxicity, metabolic syndrome

## Abstract

Heart failure with preserved ejection fraction (HFpEF) is a growing public health concern, with rising incidence alongside high morbidity and mortality. However, the pathophysiology of HFpEF is not yet fully understood. The association between HFpEF and the metabolic syndrome (MetS) suggests that dysregulated lipid metabolism could drive diastolic dysfunction and subsequent HFpEF. Herein we summarise recent advances regarding the pathogenesis of HFpEF in the context of MetS, with a focus on impaired lipid handling, myocardial lipid accumulation and subsequent lipotoxicity.

## Introduction

Across the globe, the burden of obesity is increasing at an alarming rate. Obesity rates have almost tripled since 1975, accompanied by an ever-increasing incidence of the metabolic syndrome (MetS) [1]. MetS is a constellation of metabolic conditions characterised by increased body weight, insulin resistance, dyslipidaemia and hypertension [2], that is associated with adverse cardiac remodelling, such as left ventricular hypertrophy (LVH) and stiffness. This may lead to cardiac functional impairment in the form of diastolic dysfunction, and subsequent heart failure with preserved ejection fraction (HFpEF) [3,4]. However, the exact pathophysiology of HFpEF in MetS patients is not yet fully understood. Given the close association between HFpEF and MetS, the contribution of altered serum metabolites and subsequent alterations in the uptake and use of substrates by the heart cannot be overlooked. This review will discuss the current evidence suggesting that HFpEF may be the metabolic form of heart failure, with particular focus on lipid homeostasis, and discuss the potential links between metabolic dysregulation and subsequent cardiac dysfunction, focusing on the role of lipotoxicity.

## Heart failure with preserved ejection fraction

HFpEF is a complex clinical condition in which the myocardium cannot relax sufficiently during diastole to adequately fill with blood. As a result, cardiac output is reduced, reducing perfusion of peripheral respiring tissues and thereby generating debilitating symptoms such as fatigue, exercise intolerance and dyspnoea. This trajectory is in contrast with the perhaps more well-known – and certainly better understood – form of heart failure, heart failure with reduced ejection fraction (HFrEF), in which cardiac output is reduced due to impaired myocardial contractile function during systole, most commonly secondary to myocardial damage following myocardial infarction.

### Pathological hallmarks of HFpEF

Two components broadly determine diastole and, therefore, the propensity to generate diastolic dysfunction and subsequent HFpEF. The first is the ability of the left ventricle (LV) to relax, governed by the dissociation of myofilaments which is largely dependent on cytosolic calcium dynamics. The second is the passive compliance of the LV itself, governed primarily by ventricular wall thickness, the structure of the extracellular matrix (ECM) and the particular isoform and post-translational modifications of the cardiomyocytic protein, titin. We will discuss each of these in turn, before addressing how they may be elicited by altered myocardial lipid homeostasis.

#### Intracellular calcium mishandling

Normal cardiac inotropy and lusitropy are governed by a tightly regulated cycle of calcium (Ca^2+^) release into, and reuptake from, the cardiomyocytic cytoplasm. This sequence relies on continual cycles of activation and deactivation of multiple calcium channels, such as the sarcoplasmic reticular Ca^2+^ channel (SERCA2a), the plasmalemmal sodium-calcium exchanger (NCX) and the ryanodine receptor (RyR). However, diastolic dysfunction in HFpEF is associated with impaired calcium handling. A reduction in SERCA2a function is observed in HFpEF, reducing the rate of diastolic Ca^2+^ reuptake and resulting in a state of cytoplasmic Ca^2+^ overload during diastole [5]. Both NCX and RyR functions are also modified in HFpEF, as is the function of phospholamban (PLB), exacerbating this increase in diastolic calcium load [6]. Elevated diastolic Ca^2+^ levels cause a persistent increase in actin-myosin crossbridge activation, thereby increasing resting diastolic tension and resulting in impaired cardiac filling, which characterises diastolic dysfunction [7]. The HFpEF phenotype can be reproduced to a certain extent in mice by reducing SERCA2a expression and can be rescued in both *in vitro* and *in vivo* by adenoviral and lentiviral gene transfer of SERCA2a, respectively [8–10].

#### Cardiac hypertrophy

LVH is the thickening of the LV myocardium by the addition of sarcomeres within individual cardiomyocytes. There are two types of hypertrophy: concentric, in which LV wall thickness increases significantly relative to LV chamber diameter, and eccentric, in which wall thickness to chamber diameter remains relatively constant in the presence of LV chamber dilation [11]. Concentric LVH is the most frequent structural abnormality observed in HFpEF patients [12]. This modification has been thought to be partly responsible for the diastolic dysfunction that characterises HFpEF, as haemodynamic modelling demonstrates that increased LV wall thickness increases LV end-diastolic pressure for any given LV end-diastolic volume due to increased myocardial stiffness, thus impeding LV filling [13]. In addition, inhibition of hypertrophic signalling in animal models of HFpEF improves diastolic function, and the extent of LVH is directly associated with the risk of both hospitalisation and cardiac death in HFpEF patients [14,15].

#### Extracellular matrix remodelling

The ECM is a three-dimensional network of molecules surrounding cells of a tissue that afford those cells both structural and biochemical support. Given this vital role in governing tissue architecture, changing the composition of the ECM can significantly affect tissue function. In HFpEF, it is known that myocardial fibrosis is enhanced. Human myocardial biopsies and autopsy samples demonstrate that the collagen volume fraction (CVF) of the myocardium is enhanced in HFpEF patients relative to controls [16,17]. In contrast with HFrEF, myocardial collagen deposition in HFpEF does not occur to replace lost or damaged cardiomyocytes, but instead is thought to occur as an adaptation to prevent supraphysiological sarcomeric stretch under conditions of high myocardial stress [18]. However, collagen is detrimental to diastolic function when deposited in excess as it is significantly less compliant than cardiomyocytes, thus enhances passive stiffness. As such, the extent of myocardial fibrosis is directly correlated with the extent diastolic dysfunction in HFpEF patients, and is an independent predictor of patient outcome [19,20].

#### Titin modifications

Within the I-band of striated muscle sarcomeres, myosin molecules are held in place by a giant protein called titin [21]. As a limiting factor for the range of motion of the sarcomere under tension, titin acts as a ‘molecular spring’ and is a key determinant of cardiomyocyte viscoelasticity and, by virtue, passive stiffness [22]. Titin-dependent stiffness is thought to be important in the pathogenesis of HFpEF, being increased in patients with arterial hypertension and HFpEF, but not in patients with hypertension alone [18]. In human cardiomyocytes, the shorter N2B isoform is coexpressed with its longer counterpart, N2BA, at a ratio of approximately 70:30 [23]. The N2B isoform is significantly less compliant than the longer N2BA isoform, thus one way that passive stiffness can be modulated is through titin isoform switching [24]. The ratio of N2B:N2BA isoforms is observed to increase during concentric remodelling, alongside increased passive stiffness of the myocardium [22]. Post-translational modifications of titin, particularly on the stiffer N2B isoform, have also been linked to alterations in passive stiffness, with N2B hypophosphorylation in HFpEF being associated with high diastolic stiffness [25].

### Epidemiology of HFpEF

**Figure 1:**
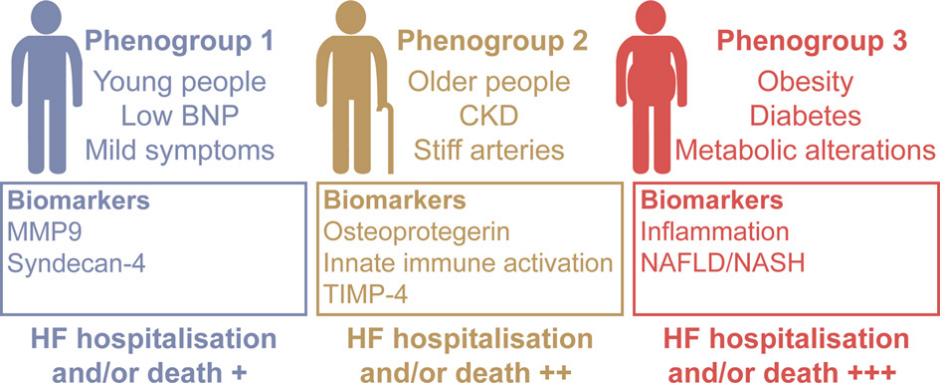
The three phenogroups of heart failure with preserved ejection fraction Data obtained from Cohen, J. et al., 2020 [31]. BNP, B-type natriuretic peptide; MMP9, matrix metalloproteinase 9; CKD, chronic kidney disease; TIMP-4, tissue inhibitor of metalloproteinase 4; HF, heart failure; NAFLD, non-alcoholic fatty liver disease; NASH, non-alcoholic steatohepatitis.

Heart failure as a whole affects approximately 0.83% of the global population; in the UK alone, more than 920,000 people are estimated to be living with heart failure, with 200,000 new diagnoses made each year [26,27]. Of these cases, HFpEF accounts for approximately 50% [28]. However, the prevalence of HFpEF is increasing 10% per decade relative to HFrEF [29]. This could in part be due to improved HFpEF diagnosis as the condition becomes more widely recognised, but is likely also due to the changing predominant aetiology of heart failure. As we improve our prevention and management of ischaemic heart disease (IHD), we would expect to see a gradual reduction in HFrEF cases, as IHD is the primary cause of this condition [26]. However, we are observing a global expansion of the ‘Western lifestyle’ and with it an increase in the prevalence of chronic metabolic disease. With this shift in morbidity to multiple chronic metabolic diseases alongside improved management of IHD, we may expect to see a relative increase in HFpEF cases. This is because HFpEF is more tightly associated with the existence of chronic conditions such as obesity, Type 2 diabetes mellitus (T2DM) and chronic kidney disease (CKD), and is a condition in which over 50% of patients have five or more co-morbidities [28]. But whilst we talk about HFpEF as one, cohesive condition, substantial heterogeneity has been found within the diagnosis of HFpEF, as three distinct ‘phenogroups’ of patients have now been identified. The first is composed of young people with low B-type natriuretic peptide (BNP) and mild symptoms; the second of older people with CKD and stiff arteries; and the third of patients with metabolic conditions, such as obesity and diabetes (Figure 1) [30,31]. This relatively wide range of aetiologies makes HFpEF an incredibly complex condition as there is no single pathophysiological mechanism unifying these groups. As such, the pathogenesis of the condition is not well characterised, limiting the identification of potential drug targets.

This difficulty in identifying drug targets for HFpEF is extremely problematic. Despite multiple trials, there are no available pharmacological agents that significantly impact outcomes in HFpEF. This presents many challenges. Studies have shown that each ambulatory HFpEF patient costs €1118 per year on average in direct healthcare expenditure in high-income countries, with each day in hospital costing $2411 [32,33]. This burden can only be expected to increase if cases are to rise alongside a lack of evidence-based therapeutics. More importantly, however, is concern over both the significant negative impact HFpEF has on patient quality of life, and the excess mortality associated with the condition. Symptoms such as dyspnoea, fatigue and exercise intolerance can drastically affect the ability of those affected by HFpEF to lead a normal life, whilst 5-year mortality estimates range between approximately 55 and 75% post-hospitalisation [34–36]. This high mortality may be due in part to the fact that there is still no clear consensus on diagnostic criteria for HFpEF, making diagnosis and follow-up of patients extremely difficult, but the lack of therapeutics is certainly a key contributor to these statistics [37]. As such, elucidation of the pathophysiological mechanisms underlying HFpEF is critical.

## The pathogenesis of HFpEF

The pathogenic mechanisms underlying HFpEF are not well characterised. The traditional theory posits that HFpEF occurs as due to pressure overload: systemic hypertension increases LV afterload, leading to LVH and subsequent diastolic dysfunction [38]. However, not all HFpEF patients suffer with hypertension. The ALLHAT trial demonstrated that patients who developed HFpEF were better identified by high body mass index (BMI) than elevated arterial pressure [39]. This suggests that pressure overload cannot be the only driving factor, and perhaps is not as important as previously thought in the pathogenesis of HFpEF. This seems likely, given the various disparate phenotypes of HFpEF patients. However, a relatively novel paradigm for HFpEF pathogenesis has been proposed with the potential to account for the pathogenesis of HFpEF in all phenogroups.

### Current paradigm of HFpEF pathogenesis

The most credited hypothesis of HFpEF pathogenesis involves coronary microvascular dysfunction (CMD) and reduced nitric oxide (NO) bioavailability. The theory proposes that the high comorbidity burden of HFpEF triggers a systemic pro-inflammatory state that impairs the physiology of the coronary endothelium, activating complex molecular pathways that converge to generate myocardial stiffening and fibrosis, thereby causing diastolic dysfunction [40]. Several studies support this. In breast cancer patients receiving radiotherapy, the relative risk of developing HFpEF increases with increasing cardiac radiation dose which, given that cardiomyocytes are resistant to radiation, suggests that coronary endothelial damage could be a precipitating factor [41]. Furthermore, in a small cohort of patients with unexplained, isolated diastolic dysfunction, parvovirus B19 was a common finding in endomyocardial biopsies and was associated with the incidence of endothelial dysfunction, suggesting that endothelial dysfunction could predispose diastolic dysfunction [42]. Looking to HFpEF patients, reduced reactive hyperaemia following suprasystolic compression of the brachial artery is observed in HFpEF patients relative to control subjects, suggestive of systemic microvascular dysfunction [43]. The PROMIS-HFpEF trial found that just under 75% of HFpEF patients demonstrate reduced coronary flow reserve (CFR), indicative of CMD, whilst Taqueti and colleagues demonstrated that impaired CFR is independently associated with worsening diastolic function [44,45]. Two further similar studies found impaired CFR and/or increased index of microvascular resistance (IMR) in 72–73% of HFpEF patients [46,47]. Together, these data suggest that HFpEF is associated with CMD, and that CMD could in fact drive the pathogenesis of HFpEF.

This hypothesis posits that CMD results in a reduction in NO bioavailability, which subsequently drives diastolic dysfunction and HFpEF. Numerous studies support this. The NO-dependent vasodilator response is impaired in the coronary microvasculature of HFpEF patients, associated with diastolic dysfunction, whilst coronary infusions of NO donors in humans acutely lowers diastolic stiffness [42,48]. Furthermore, in neonatal rat ventricular myocytes, NO attenuates the norepinephrine-induced hypertrophic response, whilst human HFpEF hearts display uncoupling of endothelial nitric oxide synthase (eNOS) associated with high myocardial stiffness and hypertrophy [49,50]. It is thought that this reduced endothelial NO bioavailability effects diastolic dysfunction by reducing the activation of guanylate cyclase in cardiomyocytes. Guanylate cyclase catalyses the conversion of guanylyl triphosphate to cyclic guanylyl monophosphate (cGMP), which then activates protein kinase G (PKG). In turn, PKG phosphorylates many proteins involved in the control of diastolic function. These include titin, which enhances diastolic distensibility; L-type calcium channels, which reduces calcium import thus reducing diastolic calcium levels; and regulator of G protein signalling, which can apply a ‘brake’ to hypertrophic signalling [51,52]. As such, reduced NO bioavailability reduces the phosphorylation of these proteins, thereby impairing diastolic function. This model is supported by *in vitro* and *in vivo* models of HFpEF in which augmenting the cGMP-PKG pathway attenuates or prevents cardiomyocyte hypertrophy, interstitial fibrosis and diastolic stiffness [49,53,54].

### Challenging the current paradigm

The aforementioned CMD paradigm of HFpEF pathogenesis hypothesises that multimorbidity induces systemic inflammation, which can in turn drive diastolic dysfunction. Given that many of the comorbidities that accompany HFpEF are associated with low-grade chronic inflammation, and that the presence of inflammatory markers is associated with CMD in a number of patient cohorts, this model appears reasonable [55–57]. Inflammation is also observed in HFpEF patients; in hypertensive patients, serum levels of CRP and TNFα were found to be associated with higher LV mass index and E:e' ratio, whilst endomyocardial biopsies from HFpEF patients show inflammatory cell infiltration that correlates with diastolic dysfunction measures [58,59]. In addition, several inflammatory biomarkers have been found to be better predictors of HFpEF severity and outcomes than natriuretic peptides [60,61]. In a recent analysis of the PROMIS-HFpEF study, inflammation was found to mediate the association between comorbidity burden and E:e' ratio, suggestive of a causative role of inflammation at least in the progression of HFpEF [62].

However, there is reason to believe that these observations are associative, and do not necessarily point to causation. While serum pentraxin-3 levels are increased in HFpEF patients, levels are highest at the coronary sinus, suggestive of its production by the coronary circulation itself, thereby suggesting that this inflammatory marker could be a consequence of dysfunction instead of a cause [63]. Serum IL-16 levels are found to correlate with the extent of diastolic dysfunction in patients. However, IL-16 neutralisation in angiotensin II-treated rats reduced myocardial fibrosis without improving diastolic dysfunction, suggesting that whilst this inflammatory marker may contribute to the HFpEF phenotype through stimulating fibrosis, it may not be crucial in the pathogenesis of the condition [64]. Further to this, it has also been observed that the early stages of HFpEF are characterised by cardiomyocyte-based passive stiffness that is not known to be driven by inflammation, with inflammation-associated fibrosis only becoming important in the later stages of disease, indicative of minimal inflammatory cell infiltration and activation of fibroblasts in early HFpEF [65]. Together, these data suggest that inflammation is an important contributor to the disease once present, but gives reason to believe that another factor could drive the initiation of the disease.

One key point to consider when searching for ‘the driving factor’ of HFpEF is that there is a high likelihood that there is no single mechanism that underlies its pathogenesis due to the aetiological and phenotypic heterogeneity of the condition. This is supported by the fact that just over 25% of HFpEF patients have no clinical signs of CMD, suggesting that CMD may not be required for the pathogenesis of HFpEF [44,46]. The results from these studies could, of course, be limited by detection thresholds for CFR and IMR measurements. However, it could also be because these studies did not account for differences in HFpEF phenogroup. Thus, it may be that CMD is critical for the pathogenesis of HFpEF in one phenogroup, whilst another mechanism is responsible for HFpEF in another.

In the particular case of the HFpEF phenogroup affected by MetS, we must consider that, whilst being a condition associated with systemic inflammation, MetS is also a condition associated with significant dysregulation in whole-body lipid metabolism. During overnutrition, adipose tissue depots expand to store excess energy. However, once the maximum capacity of adipose tissue expansion is reached, lipids ‘spillover’ into the circulation. This results in changes in both the quantity and quality of serum lipids, leading to enhanced lipid uptake by non-adipose organs, and ectopic lipid deposition. These ectopic lipids may then induce organ dysfunction through lipotoxicity. Lipotoxicity is the process by which lipid accumulation in non-adipose organs results in oxidative stress, mitochondrial dysfunction and apoptosis, leading to cellular and tissue dysfunction. This ‘lipid spillover’ from overwhelmed adipose tissue and subsequent lipotoxicity is proposed to be the mechanism underlying the pathogenesis of skeletal muscle insulin resistance, pancreatic beta-cell dysfunction and non-alcoholic fatty liver disease during MetS [66]. However, in multiple studies of systemic metabolic disease such as obesity and T2DM, the steatosis observed in these organs is mirrored by the generation of myocardial steatosis, and multiple studies of MetS patients demonstrate intramyocardial lipid accumulation [67–70]. This suggests that myocardial steatosis may be prevalent in MetS and could contribute to the pathogenesis of MetS-associated diastolic dysfunction and HFpEF.

## Myocardial lipid metabolism

### Lipid handling in the healthy heart

The heart is a metabolically demanding organ. In order to sustain continuous cardiac contraction, it requires 0.5 μmol/g/s of ATP, over 95% of which is obtained from oxidative phosphorylation [71]. Given this high demand, the human heart derives most – approximately 70–90% – of its ATP from the oxidation of energy-dense fatty acids (FAs) in the fasted state [72]. However, under different feeding states, the heart can adapt its substrate use in order to utilise the most readily available nutrients to sustain contraction. For example, in the fed state, myocardial reliance on glucose increases alongside glucose availability, governed by insulin-sensitive GLUT4-mediated glucose uptake [73]. As such, the heart has been described as a ‘metabolic omnivore’, able to use glucose, ketone bodies, branched-chain amino acids and lactate when required [74]. This metabolic flexibility allows the heart to switch between substrates in a context-dependent manner.

**Figure 2:**
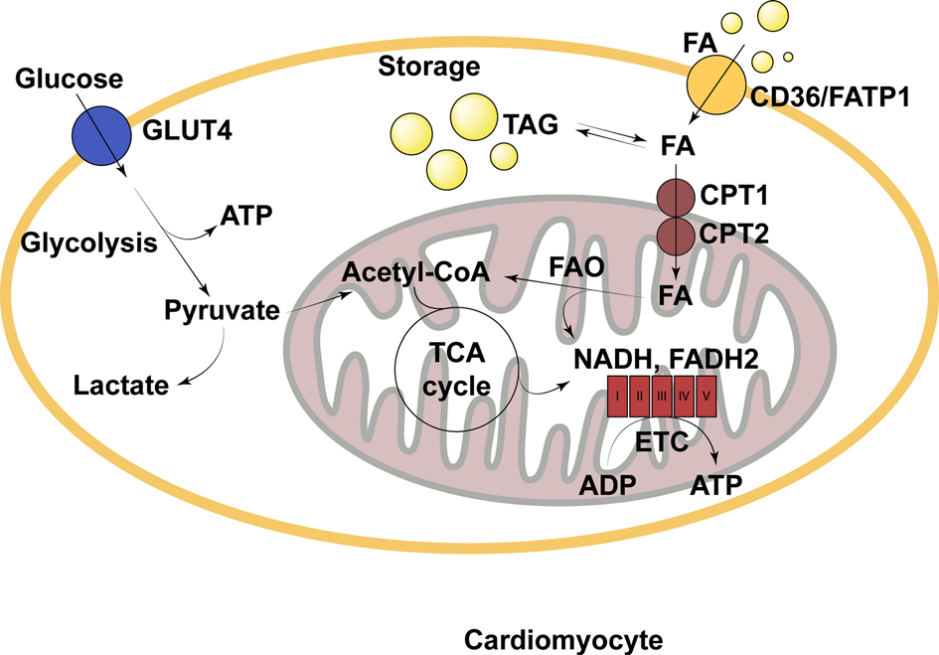
Simple schematic of fatty acid metabolism in the cardiomyocyte under physiological conditions ADP, adenosine diphosphate; ATP, adenosine triphosphate; CD36, cluster of differentiation 36; CPT, carnitine palmitoyltransferase; ETC, electron transport chain; FA, fatty acid; FADH2, reduced flavin adenine dinucleotide; FAO, fatty acid oxidation; FATP1, fatty acid transport protein 1; GLUT4, glucose transporter type 4; NADH, reduced nicotinamide adenine dinucleotide; TAG, triacylglyceride; TCA cycle, tricarboxylic acid cycle.

In order to be used to generate ATP in the heart, FAs can passively transfer across the cardiomyocytic plasma membrane, but their uptake is supported by both the FA translocase (CD36) and FA binding protein (FABP) (Figure 2). In the cytosol, FAs are predominantly converted to long-chain acylcarnitine by carnitine palmitoyltransferase I (CPT1) prior to entering the mitochondria, where they are reverted to acyl-CoA by CPT2 and enter β-oxidation. However, cytosolic FAs may alternatively be esterified to form triglycerides for storage in the myocardial triglyceride (TG) pool [72]. Despite the high reliance on FAs for ATP, total TG storage capacity within the heart is minimal, totalling only approximately 3 mg TGs stored per gram of myocardial tissue [75]. This suggests that myocardial lipid storage is tightly regulated, perhaps due to the potential for lipids to elicit adverse effects on myocardial function. However, in disease states, this tight regulation is often impaired.

### Lipid handling in HFpEF

Studies looking at substrate utilisation in the failing heart are often inconsistent. Whilst data regarding metabolism during systolic dysfunction is extensive, the data on myocardial metabolism during diastolic dysfunction are relatively minimal. Data suggest that both types of dysfunction are characterised by reduced mitochondrial oxidative metabolism, resulting in reduced ATP production, which is compensated for to some extent by increasing glycolysis [76]. During systolic dysfunction and HFrEF, myocardial fatty acid oxidation (FAO) is reduced, concordant with a generalised reduction in mitochondrial respiration [77]. However, the story is a little more complex in HFpEF. Multiple studies using surgical and genetic models of diastolic dysfunction suggest that myocardial FAO is also reduced in HFpEF [72]. However, when diastolic dysfunction is generated using models of obesity and diabetes, mimicking the MetS phenogroup of HFpEF, FAO is observed to increase [78]. This is observed in human MetS-associated HFpEF; men with diabetic cardiomyopathy exhibit increased fatty acid uptake and oxidation in association with impaired diastolic function, whilst fatty acid uptake and oxidation is increased in obese women in association with impaired myocardial efficiency [79,80]. These findings are consistent with observations in genetic models of murine obesity and diabetes, associated with LVH and diastolic dysfunction [81,82]. A comprehensive comparison of available data regarding the metabolic changes that occur in HFrEF relative to HFpEF has been performed previously [78]; as such, we will focus on HFpEF from here on in.

Increased FAO in the context of MetS-associated HFpEF likely occurs due to enhanced substrate availability. Circulating free fatty acids (FFAs) and TG-rich very low-density lipoproteins (VLDL) are increased in MetS due to the aforementioned ‘lipid spillover’ from overwhelmed adipose tissue, increasing their availability for uptake by the heart [66,83]. Serum FFAs are crucial regulators of myocardial lipid accumulation, with increased plasma FFA concentrations resulting in significantly increased myocardial FFA uptake and subsequent intramyocardial lipid deposition [84]. In addition, lipoprotein lipase (LPL) expression on cardiomyocytes is up-regulated in T2DM – a major component of MetS – enhancing VLDL hydrolysis in the coronary circulation, thereby further increasing lipid availability [85]. As such, lipid uptake by cardiomyocytes is enhanced during MetS, which likely stimulates an increase in FAO. This increase in FAO is likely further driven by reduced glucose uptake due to the downregulation of cardiomyocyte GLUT4 caused by MetS-associated insulin resistance, and by the inhibition of glucose oxidation by intermediates of lipid catabolism such as acetyl-CoA and citrate [86,87].

Despite this upregulation of FAO, evidence suggests that FAO is often insufficient to prevent myocardial TG accumulation in HFpEF. In a study using CMR to assess cardiac lipids, HFpEF patients had 2.3-fold higher myocardial lipid content than control subjects which was independently associated with diastolic strain rate [88]. Similar studies have demonstrated that HFpEF patients have significantly greater intramyocardial fat relative to both non-heart failure and HFrEF patients that independently correlates with both E:e' ratio and left atrial volume index, and that women with subclinical HFpEF have greater intramyocardial fat than reference controls [89,67]. Sharma and colleagues identified pronounced intramyocardial TG accumulation in patients with end-stage non-ischaemic heart failure, although no mention was made to whether ejection fraction was reduced or preserved in this cohort [90]. These studies demonstrate that HFpEF is associated with myocardial lipid accumulation, but could these lipids have a causative role in the pathogenesis of the condition?

## Myocardial lipotoxicity

As demonstrated by the studies listed above, HFpEF is associated with myocardial steatosis. Given that ectopic lipid accumulation in non-adipose organs is known to trigger organ dysfunction through lipotoxicity, it is plausible that myocardial steatosis may, at least in part, mediate diastolic dysfunction in this context.

### Clinical data

Several clinical studies have suggested that lipid accumulation in the heart is associated with subsequent cardiac morphological changes and diastolic dysfunction. For example, patients with congenital lipodystrophy display significant lipid accumulation in the heart in the absence of obesity, and often go on to develop hypertrophic cardiomyopathy [91]. In otherwise healthy individuals, myocardial triglyceride content is accompanied by increased LV mass on cardiac imaging [92]. In T2DM patients, consuming a very low calorie diet results in an acute increase in myocardial TG deposition associated with acute diastolic functional impairment as demonstrated by areduced E/A ratio. However, preventing lipid accumulation in this context by co-administration of the antilipolytic drug acipimox prevented this change in diastolic function, suggesting that increasing myocardial lipid deposition is sufficient to impair diastolic function [84]. Furthermore, individuals with MetS are found to have greater myocardial lipid deposits than those without, associated with impaired myocardial performance [69,70]. T2DM patients exhibit a 2.1-fold increase in myocardial TG content than normoglycaemic controls, alongside a reduction in early diastolic filling rate [68]. Together, these data show that intramyocardial fat deposition is associated with LVH and impaired diastolic function in humans. However, any causation, and the directionality of that causation, must be investigated using preclinical models.

### Preclinical models of MetS

In obese mice generated both genetically and using high-fat diet, an increase in myocardial lipid content is observed in concert with diastolic dysfunction and oxidative stress, whilst prediabetic mice exhibit diastolic dysfunction and cardiac hypertrophy in association with early changes in mitophagy and consequent cardiac lipid accumulation [93–95]. These studies demonstrate that the association between myocardial fat content and diastolic dysfunction persists in mice but do not necessarily point to causation.

### Preclinical models of altered fatty acid oxidation

Inhibition of FAO using whole-body CPT-1β-knockout mice induces myocardial lipid accumulation and exacerbates pressure overload-induced cardiac hypertrophy [96]. When FAO is impaired specifically in the heart by knockdown of PPARδ or prohibitin-2, progressive myocardial lipid accumulation is observed alongside cardiac hypertrophy, fibrosis and dysfunction [97,98]. Conversely, enhancing FAO in angiotensin II-treated, transverse aortic constricted, and high-fat diet-fed mice by a cardiac-specific deletion of acetyl CoA carboxylase 2 – relieving CPT-1 inhibition by malonyl-CoA – prevents the induction of diastolic dysfunction, alongside maintenance of myocardial energetics [99–101]. Together, these data suggest that cardiac lipid accumulation caused by impaired FAO is sufficient to induce LVH and diastolic dysfunction.

### Preclinical models of altered myocardial triglyceride lipolysis

Global genetic inactivation of adipose triglyceride lipase (ATGL) results in lipid accumulation in the heart due to a reduction in lipolytic activity, leading to LVH, myocardial fibrosis and dysfunction, and premature death [102]. This dysfunction phenotype is reversed by cardiac-specific overexpression of ATGL, suggesting that excess lipids in the heart are detrimental to cardiac morphology and function, and that dysfunction can be prevented by decreasing the amount of lipid stored [103]. Further to this, cardiac-specific overexpression of acyl-CoA synthetase results in marked cardiomyocyte TG accumulation, associated with the development of concentric LVH and dysfunction, and premature death, whilst cardiomyocyte-specific ATGL overexpression reduces stored lipids in the myocardium by increasing TG hydrolysis, and thereby improves exercise tolerance and protects from pressure overload-induced systolic dysfunction [104,105]. This demonstrates that reducing stored myocardial TGs through enhanced TG lipolysis is beneficial for cardiac morphology and function.

### Preclinical models of altered myocardial lipid uptake

GSK3α overexpression in the heart enhances myocardial FA uptake and thereby induces lipid accumulation. This results in lipotoxic cardiomyopathy, as demonstrated by enhanced fibrosis, hypertrophy and increased deceleration time [106]. Similarly, overexpressing FATP1 in the heart by placing it under the cardiac-specific MHCα promoter increases FA uptake four-fold, resulting in a two-fold increase in lipid accumulation within cardiomyocytes and associated with impaired LV filling and biatrial enlargement, indicative of diastolic dysfunction [107]. Conversely, knocking out CD36 in aged mice significantly reduces levels of myocardial lipids compared with age-matched controls due to reduced lipid uptake, associated with improved cardiac function and reduced hypertrophy [108]. This is corroborated by reducing lipid uptake by cardiac-specific deletion of LPL in αMHC-PPARα mice, which reduces myocardial lipid accumulation alongside preventing severe lipotoxic cardiomyopathy [109]. These data suggest that enhanced myocardial lipid uptake can result in myocardial lipid deposition which is associated with diastolic dysfunction, and that preventing this accumulation can prevent or reverse the cardiac phenotype.

### Contradictory evidence

The evidence presented above suggests that myocardial lipid deposition is detrimental to cardiac morphology and diastolic function, regardless of the mechanism by which the lipid accumulation occurs. Whilst the data are convincing, evidence to the contrary must also be considered. Perhaps the most obvious contradiction to this lipotoxicity hypothesis is the paradox that exists in relation to HFpEF, in which one’s risk of developing HFpEF is increased in obesity, but one's prognosis following diagnosis is improved [110]. As obesity appears protective post-diagnosis, one might suggest that lipids may not be pivotal in HFpEF. However, the fact that obesity increases HFpEF risk suggests that lipids may play a vital role in the pathogenesis of the condition. It may be that obesity precipitates HFpEF through abnormal lipid homeostasis, but subsequently improves survival post-HFpEF diagnosis as it permits the patient to withstand greater periods of cardiac cachexia, rather than being protective at a cellular level. Furthermore, we must remember that these studies do not discriminate between HFpEF phenogroups. As discussed previously, it is plausible that myocardial lipid handling alters depending on the aetiology of HFpEF, thus the effect of obesity will likely be very different in different phenogroups of HFpEF patients. However, more research is required to this end.

Further contradictory evidence comes from preclinical studies. Recent studies of the *Bscl2*^−/−^ lipodystrophic mouse model have demonstrated a reduction in myocardial lipid content in these animals [111] alongside a distinct, classical HFpEF phenotype comprising LVH, impaired active LV relaxation and increased passive diastolic LV stiffness [112]. The addition of a partial ATGL deletion in this model upregulates cardiac TG content alongside restoring cardiac function, suggestive that enhanced lipids could in fact be protective [111]. However, further metabolic analyses demonstrate that this intervention additionally improves glucose oxidation through restoration of cardiac metabolic flexibility and improves cardiac insulin sensitivity. Taken together with the findings that glucose uptake is enhanced in the hearts of *Bscl2*^−/−^ animals resulting in glucotoxicity, and that preventing this through administration of the SGLT2 inhibitor dapagliflozin reverses the HFpEF phenotype [113], this suggests that the pathogenic mechanism underlying HFpEF in this model, although metabolic, is likely not dependent on alterations in lipid homeostasis *per se*, but instead glucose homeostasis. However, these studies additionally highlight the importance of considering the potential effects that altered lipid homeostasis can have on cardiac metabolism as a whole when analysing data regarding myocardial lipid overload.

It has additionally been found that CPT-I inhibition by oxfenicine combined with a high-fat diet induces mild myocardial lipid accumulation but is not associated with LVH or changes in cardiac function [114]. However, in this study the dose of oxfenicine used was previously used to demonstrate cardiac hypertrophy and dysfunction in dogs and rats over 1 to 2 years, not 8 weeks, so is perhaps insufficient to observe an effect. Furthermore, specific indices of diastolic dysfunction were not investigated, thus we cannot conclude that diastolic function was not affected. Another study demonstrated that lipid accumulation due to high-fat feeding does not worsen cardiac morphological or functional changes in a myocardial infarction model [115]. However, ischaemic insults tend to be the driving factor for many cases of HF**r**EF, and are not usually associated with HF**p**EF. Given that lipid handling in HFrEF is distinct from that observed in HFpEF - for example, FAO is reduced in HFrEF - myocardial lipids likely have a different effect on the progression of HFrEF relative to HFpEF, thus this model may not be a good indicator of the role of lipids in HF**p**EF specifically. Together, whilst these studies suggest that we cannot state for certain that lipid accumulation is a driving factor of diastolic dysfunction, they provide insufficient contradictory evidence to rule out the role of lipids in the pathogenesis of HFpEF.

## Mechanisms underlying myocardial lipotoxicity

Together, the data outlined above demonstrate that increasing myocardial lipid storage is associated with diastolic dysfunction, whilst preventing it is sufficient to prevent functional impairments, indicating that myocardial lipotoxicity may be pivotal in the pathogenesis of HFpEF. However, it may not be TGs themselves that cause the problem. Generally, it is thought that TGs are relatively inert, thus do not mediate lipotoxicity themselves, but rather it is the accumulation of reactive lipid intermediates such as diacylglycerols (DAG), ceramides and acylcarnitines that is responsible.

Several studies support this concept (Table 1). Significant myocardial TG deposition and cardiac dysfunction is observed in a mouse model of cardiac specific PPARγ overexpression. However, when this animal is crossed with a mouse with cardiac-specific DGAT1 overexpression in order to promote lipid deposition in the form of TGs, the resultant phenotype is that of enhanced myocardial TG levels, but reduced DAGs and significantly reduced cardiac dysfunction [116]. Similarly, activation of DAG kinases to reduce DAG levels attenuates pressure overload-induced cardiac hypertrophy and dysfunction, suggestive of an important role of DAGs in the pathogenesis of this phenotype [117]. In heart failure patients, mechanical unloading of the heart by implantation of a left ventricular assist device reduces DAG and ceramide levels in the heart in concert with improved function, whilst TG levels remain unchanged [118]. In addition, echocardiographic parameters of cardiac structure and function can be improved by inhibiting ceramide synthesis in models of lipotoxic cardiomyopathy, suggesting that ceramides also play a crucial role in the pathogenesis of cardiac dysfunction [119]. In the case of acylcarnitines, the addition of cardiomyocytic PPARγ overexpression in a model of global PPARα deficiency does not alter TG, DAG or ceramide levels, but does improve cardiac function associated with reduced acylcarnitine levels [120]. In mice deficient in malonyl-CoA decarboxylase, a high-fat diet did not induce cardiac dysfunction despite enhanced TG deposition, which may be due to low levels of acylcarnitines [121]. These studies highlight the potential importance of many lipid intermediates in triggering a diastolic dysfunction phenotype, suggesting that it is not the amount of myocardial TG *per se* that mediates lipotoxic cardiomyopathy, but in fact the presence of intermediary toxic lipid species. However, the relative importance of each intermediate in triggering or worsening diastolic dysfunction remains to be established.

**Table 1:** Preclinical data on the effect of myocardial lipid intermediates on cardiac form and function

Reference	Test model	Comparator model	Effect on myocardial lipid content	Effect on survival and cardiac structure/function
Liu, L. et al., 2012 [116]	MHC-PPARγ x MHC-DGAT1	MHC-PPARγ	- Similar TAG content - Reduced DAGs and ceramides	- Improved survival - Increased fractional shortening - Trend towards reduced LV diameters
Harada, M. et al., 2007 [117]	DGK-TG + TAC	WT + TAC	- Reduced myocardial DAGs (inferred)	- Attenuated LV hypertrophy - Increased fractional shortening - Suppression of cardiac fibrosis
Park, T. et al., 2008 [119]	Lpl^GPI^ x LCB1^+/−^	Lpl^GPI^	- Similar TAG content - Reduced ceramides	- Reduced natriuretic peptide production - Improved fractional shortening
Son, N. et al., 2010 [120]	MHC-PPARγ x PPARα^−/−^	MHC-PPARγ	- Similar TAG content - Reduced acylcarnitines	- Improved survival - Increased fractional shortening
Ussher, J. et al., 2009 [121]	MCD^−/−^ + DIO	WT + DIO	-Increased TAG content - Reduced long-chain acylcarnitines	- Reduced LV posterior wall thickness

Together, these studies suggest that reducing myocardial lipid intermediate content improves cardiac function and survival. However, data on specific diastolic function measures are not included in the data presented in these studies, likely due to the relatively understudied nature of this condition. DAG, diacylglycerol; DGAT, diacylglycerol acyltransferase; DGK, diacylglycerol kinase; DIO, diet-induced obesity; GPI, glycosylphosphatidylinositol, represents membrane-anchored overexpression; LCB1, serine palmitoyltransferase subunit 1; Lpl, lipoprotein lipase; MCD, malonyl CoA decarboxylase; MHC, myosin heavy chain, represents cardiac-specific overexpression; PPAR, peroxisome proliferator activated receptor; TAC, transverse aortic constriction; TAG, triacylglycerol; TG, transgenic; WT, wild-type.

There are numerous pathways by which lipid intermediates can elicit deleterious effects in cardiomyocytes that lie outside the scope of this review but have been excellently reviewed elsewhere [75]. However, two key players – namely the production of excess reactive oxygen species (ROS) and mitochondrial dysfunction – are outlined below.

### Enhanced reactive oxygen species production

Myocardial steatosis is known to enhance ROS production: multiple models of cardiac lipid accumulation – such as ATGL deficiency, palmitate treatment, overexpression of ceramide synthase and genetic models of obesity and diabetes – all result in increased ROS production [122,123,75]. Lipid intermediates may be responsible for this, as both DAGs and ceramides upregulate NADPH oxidase (NOX2) via activation of protein kinase C (PKC), leading to enhanced ROS production [124,125,86]. It has also been argued that increased FAO may contribute to the ROS burden, as FAO is less oxygen efficient than glucose oxidation, leading to the production of mitochondrial ROS [75]. However, several experimental models that increase FAO do not adversely affect heart function unless there is concurrent inappropriate lipid accumulation (see ‘Preclinical models of altered fatty acid oxidation’), suggesting that this may not be a significant source of ROS.

**Figure 3:**
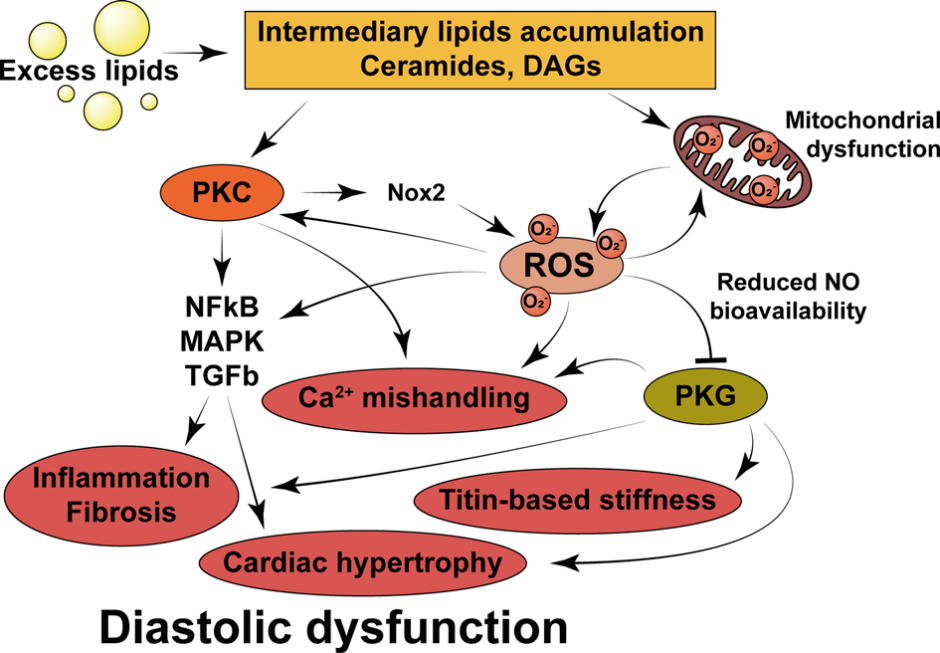
Mechanisms by which lipid intermediates can promote the generation of reactive oxygen species (ROS), and by which ROS may go on to elicit diastolic dysfunction Physiological lipid handling is detailed in Figure 2 for comparison. PKC, protein kinase C; DAGs, diacylglycerols; Ca^2+^, calcium; PKG, protein kinase G; Nox2, nicotinamide adenine dinucleotide phosphate oxidase 2; NF-kB, nuclear factor kappa-light chain enhancer of activated B cells; MAPK, mitogen activated protein kinase; TGFb, transforming growth factor beta; O^2−^, superoxide anion radical.

Once produced, ROS can affect myocardial function in a number of ways (Figure 3). ROS can deplete NO bioavailability by diverting NO to peroxynitrite through reaction with superoxide. HFpEF myocardium displays increased nitrotyrosine staining, suggestive of enhanced diversion of NO to form peroxynitrite [51]. This could then contribute to the pathogenesis of HFpEF through a reduction in NO-dependent cGMP-PKG signalling, as described previously (see ‘Current paradigm of HFpEF pathogenesis’). In addition, peroxynitrite activates protein phosphatase 2a activity, which lowers PLB phosphorylation, reduces sarcoplasmic reticular Ca^2+^ uptake, and increases diastolic cytosolic Ca^2+^, all of which can contribute to calcium mishandling in HFpEF [126]. ROS are also able to activate the RyR on the sarcoplasmic reticulum, enhancing calcium release into the cytosol. Whilst this is thought to be an adaptive mechanism to enhance RyR activity under conditions of increased demand, chronic activation enhances cardiac passive tension through increased calcium-dependent cross-bridge activation, which could represent a mechanism underlying HFpEF pathogenesis [127]. This is a vicious cycle, as it is known that enhanced calcium release from the sarcoplasmic reticulum can enhance ROS production through NOX2 activation, further adding to the ROS burden [125]. Furthermore, ROS have been shown to activate a variety of hypertrophic and pro-fibrotic signalling pathways in the heart, such as NF-κB and MAPK, as well as to enhance disulphide bonding on the titin N2B isoform, enhancing myocardial stiffness and thereby contributing to the pathogenesis of diastolic dysfunction [128,129].

ROS can also potentiate lipid intermediate-induced PKC activation due to the redox-sensitivity of PKCs [130]. In turn, PKC signalling can modify multiple pathways involved in regulating diastolic function, independently of ROS, as alluded to by the fact that overexpression of various PKC isoforms leads to heart failure [86]. For example, this pathway has been shown to activate fibroblasts resident in the myocardium as well as to increase NF-κB and TGFβ signalling, inducing the production and deposition of excess ECM components and thereby promoting the development of fibrosis [131,132]. PKC signalling is also associated with negative inotropy, impaired calcium handling, increased cardiomyocyte necrosis and enhanced ventricular wall thickening [86].

### Mitochondrial dysfunction

Mitochondrial dysfunction is widely observed in heart failure, as it is in studies of cardiac lipotoxicity [133]. In ATGL-KO mice, mitochondria are enlarged, but mitochondrial number is significantly reduced, whilst inhibition of mitophagy during high fat diet-feeding induces mitochondrial dysfunction and exacerbates cardiomyopathy [103,134]. Mitochondrial uncoupling – the phenomenon by which the electron transport chain is uncoupled from ATP production – is associated with cardiac lipotoxicity, and mitochondrial homogenates from steatotic hearts demonstrate reduced basal and stimulated oxygen consumption relative to wild-type, suggesting that mitochondrial function is impaired by myocardial lipid accumulation [135,103]. Furthermore, lipoprotein lipase knockdown in mice with cardiac-specific PPARα overexpression rescues cardiac function, associated with reactivation of transcriptional regulators of mitochondrial function and improvements in mitochondrial ultrastructure [109]. This shows that myocardial lipid accumulation can induce mitochondrial dysfunction, and that this could represent one mechanism by which the lipotoxic effects of excess lipids are mediated in the heart.

It may be that the ROS produced by excess lipid intermediates are responsible for mitochondrial damage and dysfunction in this context. Reducing lipid-induced ROS production by inhibiting NOX2 prevents mitochondrial dysfunction in cardiomyocytes treated with palmitate *in vitro*, suggesting that ROS can damage mitochondria themselves [125]. This may be through effects on the mitochondrial membrane: both ROS and peroxynitrite can target mitochondrial membrane phospholipids such as cardiolipin to generate lipid peroxidation products, which have been observed to accumulate in heart failure and are known to induce mitochondrial uncoupling and calcium overload [136]. This is problematic, as mitochondrial calcium homeostasis is critical for mitochondrial function; any deviations from optimal levels can result in reduced metabolic enzyme activity or activation of cell death pathways [77]. The mitochondrial membrane may additionally be altered directly by excess lipids themselves. It has been suggested that when the supply of FAs overwhelms the oxidative and storage capacity of the cardiomyocyte, excess saturated FAs are incorporated into cardiomyocyte membrane phospholipids, which may contribute to the development of diastolic dysfunction through altered membrane fluidity, down-regulation of cardiac ion channels and altered calcium fluxes [137–140]. However, this incorporation has been shown to occur not only in the cardiomyocyte membrane itself but also in the mitochondrial membrane, precipitating mitochondrial dysfunction [141].

**Figure 4:**
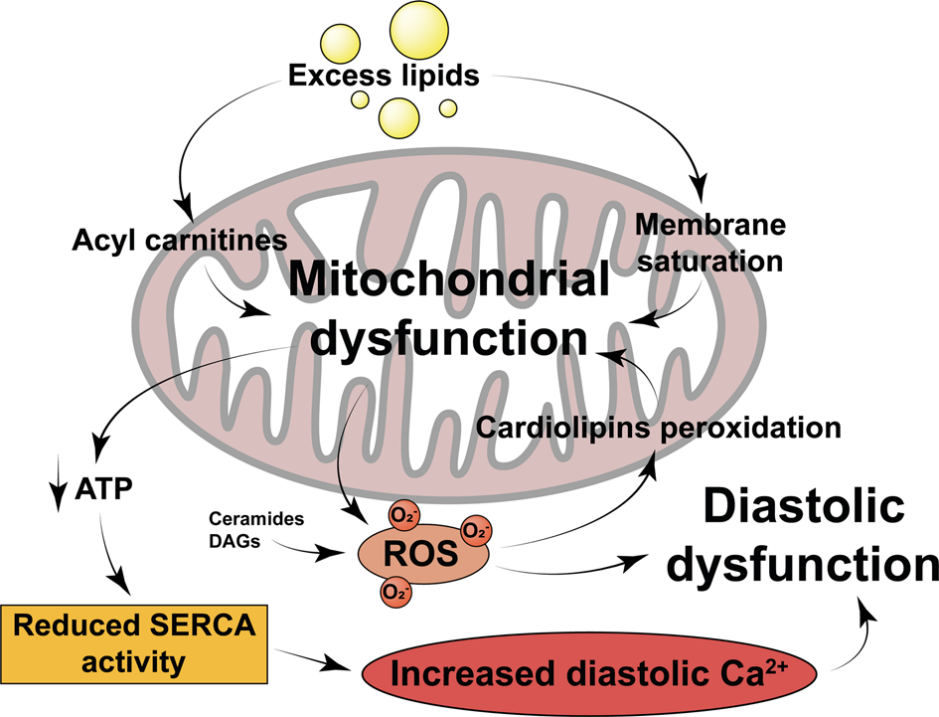
Mechanisms by which lipid intermediates may induce mitochondrial dysfunction, and by which mitochondrial dysfunction may mediate diastolic dysfunction Physiological lipid handling is detailed in Figure 2 for comparison. ATP, adenosine triphosphate; ROS, reactive oxygen species; SERCA, sarco/endoplasmic reticulum calcium ATPase; Ca^2+^, calcium; O^2−^, superoxide anion radical.

Mitochondrial dysfunction could be responsible for wider myocardial dysfunction in two key ways (Figure 4). First, it is known that mitochondrial dysfunction increases the generation of mitochondrial ROS which can damage cellular components and cause subsequent dysfunction, as discussed previously. In muscle cells, ceramides induce mitochondrial ROS production through mitochondrial fission, whilst long-chain acylcarnitines have been shown to acutely increase mitochondrial ROS, adding to the pre-existing ROS burden [142,143]. Further to this, angiotensin II-induced cardiomyopathy can be rescued by reducing mitochondrial ROS using mitochondrial-targeted antioxidant peptides, suggesting that mitochondrial ROS could be significant in potentiating cardiac dysfunction [144]. However, functional impairment could also be mediated by impaired cardiac energetics. As the heart acquires the majority of its high demand for ATP from mitochondrial oxidative phosphorylation, mitochondrial dysfunction and impaired energetics could lead to cardiac dysfunction. Pressure overload-induced heart failure is associated with significantly impaired cardiac oxidative capacity, mainly due to mitochondrial defects [145]. Furthermore, the enhanced mitophagy and reduced mitochondrial biogenesis observed in heart failure could reduce mitochondrial numbers, thereby potentially reducing the oxidative capacity of the cell further [77]. Correspondingly, the ratio of phosphocreatine to ATP – an indicator of myocardial energy reserve – is significantly reduced in HFpEF [146]. This reduction in ATP availability may be mediated by mitochondrial dysfunction but could also occur as a direct result of increased myocardial lipid deposition. Futile cycling of FAs between the intracellular TG pool and their acyl-CoA moieties utilises ATP unnecessarily, whilst long-chain FAs can activate membrane Ca^2+^ channels, increasing non-contractile ATP hydrolysis due to the need to actively maintain Ca^2+^ homeostasis [75,147]. Together, this ATP depletion may contribute to the pathogenesis of HFpEF through reducing ATP-dependent calcium channel activity, resulting in enhanced cross-bridge activation during diastole and increased diastolic pressure [148].

## Relating lipotoxicity to the current paradigm

The weight of the evidence supporting the current paradigm of HFpEF pathogenesis – that systemic inflammation induces CMD, which reduces NO bioavailability and induces diastolic dysfunction – cannot be ignored. However, when combined with the evidence presented on the contribution of lipotoxicity to the pathogenesis of cardiac dysfunction, we must ask ourselves whether the two paradigms may be linked, particularly given that myocardial steatosis has been shown to be associated with CMD [67]. In the proposed paradigm by Paulus and Tschope, NO bioavailability is specifically reduced in endothelial cells (ECs) [40]. However, rather than being a product of systemic inflammation, this could be due to the effect of excess circulating FFAs/VLDLs on ECs, in concert with cardiomyocytic steatosis.

Conditions of high circulating FFAs are associated with endothelial dysfunction [149]. TG-enriched HDL is independently associated with the extent of brachial arterial dysfunction, which may be indicative of widespread endothelial dysfunction, whilst coronary endothelial dysfunction – as assessed by reactive hyperaemia index during coronary angiography – is directly and independently associated with LDL:HDL ratio [150,151]. These data suggest that lipids could mediate CMD. This is likely driven by a reduction in NO bioavailability, given that *in vivo, in vitro* and clinical studies have all shown that elevated FFAs reduce NO bioavailability in ECs [152].

The mechanisms by which lipids reduce NO bioavailability in ECs are varied. Excess lipids can significantly increase endothelial ROS production, which can then react with NO to form peroxynitrite, depleting NO supply [152]. ROS can also deplete the NOS cofactor, tetrahydrobiopterin, further reducing NO bioavailability [153]. Elevated circulating FFAs are also associated with a 53% reduction in eNOS activity in the rat aortic endothelium, which may be mediated by FA-stimulated *de novo* synthesis of DAGs and ceramides in ECs, both of which activate PKC and thereby inhibit eNOS signalling [154–157]. Excess FAs are also known to induce insulin resistance through PKC-mediated downregulation of cell surface receptors, which reduces insulin-stimulated eNOS activation in ECs, thus demonstrating yet another mechanism by which excess FFAs can reduce NO bioavailability [152]. These studies demonstrate that lipids can induce CMD through reducing NO bioavailability in ECs, mirroring the current paradigm of HFpEF pathogenesis, but doing so independently of the systemic inflammation proposed as a driver by Paulus and Tschope.

## Conclusion

Whilst the mechanisms underlying the pathogenesis of HFpEF remain contentious, impaired lipid handling and subsequent lipotoxicity in both cardiomyocytes and coronary ECs play a key role in mediating diastolic dysfunction. It is likely that these processes drive the CMD and reduced NO bioavailability described in the current paradigm, particularly in the context of MetS, alongside exerting myriad detrimental effects on cardiomyocytes themselves. Whether lipids drive this phenotype independently of, or in concert with, systemic inflammation remains to be established, but these data demonstrate that lipids must be considered as key players in the pathogenesis of HFpEF. Efforts must now be made using *in vitro* models to investigate the particular lipid species that exert cytotoxic effects, and the precise cellular mechanisms underlying this dysfunction, with a focus on oxidative stress and subsequent mitochondrial dysfunction. Further studies will also be required to establish the interplay between lipids and inflammatory mediators in this context. In light of this, future therapeutic strategies should seek to improve cardiomyocyte lipid handling as a method by which to prevent or ameliorate HFpEF, particularly in the context of the MetS phenogroup. This may include strategies to enhance lipid use, to limit myocardial lipid uptake, or to ensure that lipid is stored as ‘inert’ species. Many previous studies have not shown great promise for these strategies, but this is likely due to a lack of focus on this particular form and aetiology of heart failure. As such, we must first focus on defining the hallmarks of the MetS phenogroup of HFpEF patients and strive to generate murine models that accurately mimic the metabolic and cardiovascular phenotype observed in this condition. Without this, we run the risk of discounting potential therapeutics due to their lack of efficacy in other phenogroups, thereby delaying the identification of much-needed treatments for patients suffering from MetS-associated HFpEF.

## Data Availability

Data sharing is not applicable to this article as no new data were created or analyzed in this study.

## Open Access

Open access for this article was enabled by the participation of University of Cambridge in an all-inclusive *Read & Publish* pilot with Portland Press and the Biochemical Society under a transformative agreement with JISC.

## Abbreviations

ADP: adenosine diphosphate
ATGL: adipose triglyceride lipase
ATP: adenosine triphosphate
BMI: body mass index
BNP: B-type natriuretic peptide
Ca^2+^: calcium
CD36: cluster of differentiation 36 (also known as FA translocase)
CFR: coronary flow reserve
cGMP: cyclic guanosine monophosphate
CKD: chronic kidney disease
CMD: coronary microvascular dysfunction
CPT: carnitine palmitoyltransferase
CRP: C-reactive protein
CVF: collagen volume fraction
DAG: diacylglycerol
DGAT: DAG acyltransferase
DGK: DAG kinase
DIO: diet-induced obesity
EC: endothelial cell
ECM: extracellular matrix
eNOS: endothelial NO synthase
ETC: electron transport chain
FA: fatty acid
FABP: FA binding protein
FADH2: reduced flavin adenine dinucleotide
FAO: fatty acid oxidation
FATP1: FA transporter protein 1
FFA: free fatty acid
GLUT4: glucose transporter 4
GPI: glycosylphosphatidylinositol
GSK3a: glycogen synthase kinase 3 alpha
HDL: high-density lipoprotein
HF: heart failure
HFpEF: heart failure with preserved ejection fraction
HFrEF: heart failure with reduced ejection fraction
IHD: ischaemic heart disease
IL-16: interleukin 16
IMR: index of microvascular resistance
KO: knockout
LCB1: serine palmitoyltransferase subunit 1
LDL: low-density lipoprotein
LPL: lipoprotein lipase
LV: left ventricle
LVH: left ventricular hypertrophy
MAPK: mitogen-activated protein kinase
MCD: malonyl CoA decarboxylase
MetS: metabolic syndrome
MHC: myosin heavy chain
MMP: matrix metalloproteinase
NADH: reduced nicotinamide adenine dinucleotide
NAFLD: non-alcoholic fatty liver disease
NASH: non-alcoholic steatohepatitis
NCX: sodium-calcium exchanger
NF-kB: nuclear factor kappa-light chain enhancer of activated B cells
NO: nitric oxide
NOX2: NO oxidase
O^2-^: superoxide anion radical
PKC: protein kinase C
PKG: protein kinase G
PLB: phospholamban
PPAR: peroxisome proliferator-activated receptor
ROS: reactive oxygen species
RyR: ryanodine receptor
SERCA2a: sarco/endoplasmic reticulum calcium ATPase
SGLT2: sodium glucose cotransporter 2
T2DM: type 2 diabetes mellitus
TAC: transverse aortic constriction
TAG: triacylglycerol
TCA Cycle: tricarboxylic acid cycle
TG: triglyceride
TGFb: transforming growth factor beta
TIMP: tissue inhibitor of MMP1
TNFa: tumour necrosis factor alpha
VLDL: very low-density lipoprotein
WT: wild-type
